# Supplementary material for: Identifying widespread and recurrent variants of genetic parts to improve annotation of engineered DNA sequences
Source: PLoS One. 2024 May 28;19(5):e0304164. doi: 10.1371/journal.pone.0304164 (PMC11132462; doi:10.1371/journal.pone.0304164)
Supplement: Attachment — Submitted filename: Genetic Part Variants - Response Letter - 2024-03-28.pdf [file pone.0304164.s002.pdf]

We thank the editorial team and both reviewers for their constructive comments and have responded below in blue text.

Editorial Comments

1. Please ensure that your manuscript meets PLOS ONE's style requirements, including those for file naming. The PLOS ONE style templates can be found at

https://journals.plos.org/plosone/s/file?id=wjVg/PLOSOne_formatting_sample_main_body.pdf and

https://journals.plos.org/plosone/s/file?id=ba62/PLOSOne_formatting_sample_title_authors_affiliations.pdf

We read these documents and updated the manuscript to be in accordance with PLOS ONE's style requirements.

2. We note that you have referenced (Raciti D, Yook K, Harris TW, Schedl T, Sternberg PW. Micropublication: incentivizing community curation and placing unpublished data into the public domain. Database. 2018;2018: bay013. doi:10.1093/database/bay013) which has currently not yet been accepted for publication. Please remove this from your References and amend this to state in the body of your manuscript: (ie "Bewick et al. [Unpublished]") as detailed online in our guide for authors

http://journals.plos.org/plosone/s/submission-guidelines#loc-reference-style

We are unsure how this citation was classified as unpublished by your article check? It shows a status of **Published: 02 March 2018** on the *Database* journal website:

- https://academic.oup.com/database/article/doi/10.1093/database/bay013/4917853
- https://doi.org/10.1093/database/bay013

Therefore, we did not make this change, and left this citation as it was.

Reviewer Comments

Reviewer #1: This manuscript addresses a common problem in molecular biological engineering communities about how well we know our plasmids. Using pLannotate pipeline that Author's lab recently developed, Authors discovered that there are uncharacterised genetic variations in the plasmids deposited in public depository database. The manuscript describes a finding in DNA sequence evolution in laboratories, which has been ignored broadly by the communities. This finding may explain a degree of phenotype variations in the characterisation works among different laboratories. Overall, this manuscript is acceptable.

However, it would be nice to address the following comment:

(1) Could Authors cut the result session into several sub-sessions to address a specific argument point per sub-session? In the current version, there is only one session-the whole result, but five figures. It is quite difficult to read through the whole result session with a clear mind.

We added subheadings to the Result to conceptually divide it up and make it more digestible.

Reviewer #2: In this manuscript, the authors use a bioinformatics approach to analyse a large collection of cloning and expression vectors to investigate variation in the different 'genetic parts' (i.e.

components of the vector). Their overall motivation is to identify different part variants, so as to enable such variants to be annotated in plasmid sequences, and to signpost future research to test whether this variation affects the properties of that part. The research is valuable and thoughtfully done, and my suggestions are relatively minor:

- Figure 1. Please check the legend, since it appears to be incorrect (e.g. referring to orange triangles). I was also a bit confused by the distinction between 'total variants' and 'unique variants' — I assume that the latter refers to variants represented only once in the dataset? Or does 'total variants' imply some higher-level classification e.g. promoter types (Plac, PrrnB1 etc), and 'unique variants' refer to variants within this perhaps varying by only single bp — but in this case why are there fewer unique variants than total variants? Some clarification would be beneficial, and perhaps a cartoon describing the process would be a good way of doing this.

Thank you for the correction and the request for clarification.

We fixed color/symbol mismatches between this figure, its legend, and the text.

By "unique variants" we meant "distinct variant sequences". That is, if the same change of just one base from the canonical sequence is observed in 200 examples of a genetic part across different plasmids, that counts 200 times for "total variants" and 1 time for "distinct variant sequences". Figure one sums these values over all examples of parts classified into the different categories.

We recognize how the phrasing "unique variants" could also be interpreted as variants occurring once in the entire dataset. To clarify what we are showing in the results, we changed that imprecise description throughout the text and in the figures to "distinct variant sequences".

For example, we made this change in the Fig. 1 legend:

> Within each part type, the total number of genetic parts (green squares), total number of genetic parts that are variants (i.e., differ from the canonical sequence) (orange circles), and number of distinct genetic part variant sequences (i.e., counting each unique sequence that differs from the canonical sequence one time) (blue triangles) are plotted.

- Line 255. Was it exactly 7,500,000 pairwise comparisons, and why was this number chosen?

No, not exactly. This number was rounded from the 7,508,114 pairwise comparisons we used in our authorship analysis. It is determined by the sets of all pairwise comparisons of plasmids containing each part variant for all part variants that were observed < 1205 total times in the overall dataset (the variants to the left of the orange line in **Fig. 2B**). It maxed out at this value for practical computing reasons. As the number of plasmids with the part variant grows, the number of all-versus-all comparisons between them grows rapidly.

To better convey this information, we altered this part of the legend to read:

> The distributions of DS scores and percent identities for pairwise comparisons of plasmids that share undocumented part variants are plotted. Every plasmid containing a given genetic part variant that was observed 1205 or fewer total times was compared to every other plasmid with that part variant for a total of 7,508,114 comparisons.

- It would be interesting to construct and visualise phylogenetic trees for some of the key components that have shown potential evolution, but this may not be possible, and is certainly not necessary for the current manuscript.

We agree that this would be very interesting! It is why we started this line of research in the first place. We hope to follow up with more detailed analyses in future work, including phylogenetic trees and functional tests, for variants of certain parts that exhibit interesting patterns/histories.
